# 

**Published:** 1858-08

**Authors:** 


☞ The Toledo Blade deliberately perpetrated the following:

The Dentists of Northern Ohio met in Convention yesterday, in Cleveland. They were to discuss several interesting questions. If they don't *jaw* too much, and push their investigations in the *teeth* of opposition, much truth may be *drawn out* in this meeting, which might otherwise pain the possessors in decaying, and to this end it is to be hoped that the Dentists will "*pull* all together."

---

☞ A lady in England, nearly eighty years of age, who was for many years toothless, has cut an entire row of new teeth, causing her intense suffering.

### CURRENCY BANKABLE IN CINCINNATI.

| | | |
|---|---|---|
| ALABAMA—Southern Bank of Mobile | | 1 per cent. dis. |
| | Bank of Mobile | 1½ " dis. |
| | All others | from 5 to 10 " dis. |
| CANADA—All 1 per cent. discount except Colonial Bank | | no sale. |
| | Zimmerman Bank, Elgin | 10 per cent. dis. |
| CONNECTICUT—Par, except Bank of Hartford Co. | | 2 " dis. |
| | " Litchfield County | 2 " dis. |
| | " North America | 10 " dis. |
| | Hatters' Bank, Bethel | 1 " dis. |
| | Litchfield Bank, Litchfield | 50 " dis. |
| | Pequonnock Bank, Bridgeport | 2 " dis. |
| | Pilgrims' Harbor Bank, W. Meriden | no sale. |
| | Uncas Bank, Norwich | 2 per cent. dis. |
| | Woodbury Bank, Woodbury | 50 " dis. |
| | Wooster Bank, Danbury | 2 " dis. |
| DELAWARE—Par. | | |
| DISTRICT OF COLUMBIA—All 1½ discount, except corporation of Washington, Alexandria, and Georgetown | | 10 per cent. dis. |
| GEORGIA—All from 2 to 5 per cent. discount, except Bank of the Empire State | | 10 " dis. |
| | Cherokee Insurance and Banking Co. | 15 " dis. |
| | Exchange Bank, Griffin and Savannah | no sale. |
| | Timber Cutters' Bank, Savannah | " |
| ILLINOIS—All 1½ per cent. discount, except Bank of Brooklyn | | " |
| | Bank of Prairie du Chien | " |
| | Cairo City Bank | " |
| | Continental " | " |
| | Darien Stock " | " |
| | Garden City " | " |
| | Manufacturers' and Exchange Bank | " |
| | Narraganset Bank | " |
| INDIANA—All ½ to 1 per cent. discount, except Bank of the State | | Par. |
| | State Bank | Par. |
| | Bank of Gosport | 3 per cent. dis. |
| | Merchants and Mechanics' Bank | 3 " dis. |
| | Union Plank Road Co. | 10 " dis. |
| IOWA—All 50 to 75 per cent. discount. | | |
| KANSAS—Citizens' Bank Checks on New York | | Par. |
| | Valley Bank | 50 per cent. dis. |
| LOUISIANA—Par, except Bank of Commerce | | no sale. |
| KENTUCKY—Par. | | |
| MAINE—Par, except Umbeguagus Bank, Bangor | | no sale. |
| | Bank of Hallowell | Broke. |
| | China Bank | Broke. |
| MARYLAND—Baltimore Banks | | Par. |
| | Country Banks | ½ to 3 per cent. dis. |
| MASSACHUSETTS—All par. | | |
| MICHIGAN—All 1 per cent. discount, except Bank of Macomb Co. | | 75 per cent. dis. |
| | Bank of Tecumseh | 5 " dis. |
| MISSOURI—Par. | | |
| NEBRASKA—All from 50 to 75 per cent. discount. | | |
| NEW BRUNSWICK—All 6 per cent. discount. | | |
| NEW HAMPSHIRE—Par. | | |

New Jersey—Par, except Morris' County Bank,..................................3 per cent. dis.
Ocean County Bank,.............................................3 " dis.
Stocks Security,..................................................3 " dis.
New York—City Bank par. All others par except Bank of Central New York,....3 " dis.
Bank of Ellicott,.......................no sale.
Bellinger Bank,.......................no sale
Dairyman's Bank,..............5 per cent. dis.
Otseningo Bank,.... ..........5 " dis.
Tioga County Bank,...........5 " dis.
North Carolina—All 2½ per cent., except Farmers' Banks,.....................5 " dis.
Nova Scotia—All ten per cent.
Ohio—Par, except Union Bank of Sandusky City,..................................5 " dis.
City Bank of Columbus,.........................................5 " dis.
City Bank of Cincinnati,......................................30 " dis.
Seneca County Bank and Canal Bank, Cleveland,...........75 " dis.
Pennsylvania—Philadelphia Banks par, except Commercial Exchange Bank,............no sale.
Union Bank,............................ "
Pittsburg Banks,..........................Par.
Country Banks, from 1 to 10 per cent discount.
Rhode Island—Par, except Smithfield Lime Rock Bank,...............................no sale.
Warwick Bank,..................................10 per cent. dis.
South Carolina—All 1 per cent. discount.
Tennessee—Bank of Tennessee,...... ............................................2 per cent. dis.
Planters' Bank,..............................................2 " dis.
Union Bank,....................................................2 " dis.
All others from 3 to 50 per cent. discount, as the rates vary every day.
Vermont—Par.
Virginia—Wheeling City Banks,..........................................................Par.
All others, 1 to 2 per cent. discount.
Wisconsin—All 10 per cent. discount, except Bank of Manitowoc,......................no sale.
Buffalo County Bank,...................... "
Citizens' Bank of Oshkosh,................ "
City Bank of Prescott,..................... "
Ko koma Bank, ............................. "
La Cross Bank,............................. "
Marathon City Bank,...................... "
Menominee Bank........................... "
Northern Wisconsin Bank,................ "
St. Louis Bank,............................ "
Osborne Bank,.............................. "
Southern Bank,............................. "

### CINCINNATI MARKET.

August 19th 1858.

Flour—Demand Active. A further advance has been established. Sales of 1100 bbls, $4.85@$4.87 for good superfine. Receipts for 24 hours. 1743 bbls.

Whiskey—In good demand. Sales lar[illegible], but market not as buoyant. Sales add up 1900 bbls. at 23¼@23½—including 150 bbls. old at 24¼ cents.

Provisions.—Market quiet, with some indication of a decline. Only sales to day we heard of were 100 bbls.[illegible]ess pork, at $16.50.

Groceries—Molasses is unsettled, and, except in a small way, can not be bought below 45c, holders anticipating a further advance. Sugar is firm, 8¼@9½, with a fair demand. Coffee, 11@12c. and in moderate demand.

GRAIN—Wheat—The market opened buoyant, and for good white prices were a shade better. Sales 950 Bush., prime white at $1.06 200 good white at $1 *delivered*. Receipts for 48 hours, 11,614 bush.

Corn—Market declining, with a good demand, at 55c. Received past 48 hours, 5837 bushels.

Rye—Demand fair. Prices firm. at 65c.

Barley.—Prices for old have advanced, with a good demand. Holders are not disposed to sell at present. One lot of 1000 bush. sold at 60c. No sales of new.

Oats—Market firm, with a good demand, at 52@53c. Receipts large, but holders are storing them in anticipation of higher prices. Receipts for 48 hours, 9098 bush.

Butter.—Demand good and prices firm, at 12@13c. for good and prime, and 14@15c. for choice and extra.

Cheese—A good demand and market firm, at 6¼c. for selected W. R., and 6¾@7c. for extra large.

Potatoes—Demand good and prices steady, at $2@$2.25 per bbl. We heard of no sale except by private contracts.

Fruit—The inquiries for dried apples are numerous, and prices steady, at $1.75@$1.87 per bushel. There are no prime dried peaches in the market, and prices nominal, at $4.50

Eggs—Demand fair and prices firm, at 6@7 for unselected and 8c. for selected.

